# miR-12 and miR-124 contribute to defined early phases of long-lasting and transient memory

**DOI:** 10.1038/s41598-017-08486-w

**Published:** 2017-08-11

**Authors:** Julia Michely, Susanne Kraft, Uli Müller

**Affiliations:** Biosciences Zoology/Physiology-Neurobiology, ZHMB (Center of Human and Molecular Biology) Faculty NT – Natural Science and Technology, Saarland University, D-66123 Saarbrücken, Germany

## Abstract

MicroRNAs (miRNAs) are important epigenetic regulators of mRNA translation implicated in long-lasting synaptic plasticity and long-term memory (LTM). Since recent findings demonstrated a role of epigenetic regulation of gene expression in early memory phases we investigated whether epigenetic regulation by miRNAs also contributes to early memory phases. We used the olfactory associative learning paradigm in honeybees and addressed the contribution of miRNAs depending on the conditioning strength. We selected miR-12, miR-124, and miR-125 that have been implicated in processes of neuronal plasticity and analysed their contribution to non-associative and associative learning using miRNA inhibitors. Blocking miR-12, miR-124, or miR125 neither affects gustatory sensitivity nor habituation nor sensitization. Blocking the function of miR-12 and miR-124 during and shortly after 3-trial conditioning impairs different early memory phases. Although different, the function of miR-12 and miR-124 is also required for early phases of transient memory that is induced by 1-trial conditioning. Blocking miR-125 has no effect on early memory independent of the conditioning strength. These findings demonstrate that distinct miRNAs contribute to early phases of both, transient memories as well as long-lasting memories.

## Introduction

MicroRNAs (miRNAs) are short non-coding RNA molecules (21–23 nt). They exert regulatory functions by direct RNA-RNA antisense interaction with their target messengerRNA (mRNA)^1^. miRNAs are engaged in numerous physiological processes including maturation, connectivity and plasticity of neurons^2^ and are known to be modifiers in learning and memory processes in both vertebrates and invertebrates^3–6^. Well known players in synaptic plasticity and learning processes like the transcription factor CREB and translation regulator CPEB are modulated by miRNAs^7, 8^. In mice lack of the endoribonuclease *Dicer1* results in an enhanced cognition due to decreased levels of mature miRNA^9^. Supportingly, elevated miRNA levels by overexpression result in impaired memory and synaptic plasticity^4, 10, 11^ and impairments in spatial memory^12^. In rats, memory impairment resulting from elevated miRNA levels was shown to occur within specific time-windows^13^. While these findings show that artificial elevation of miRNA levels suppresses memory formation, other studies demonstrate that conditioning induce a fast elevation of miRNA levels that is essential for memory formation. Blocking the function of conditioning-induced elevation of miRNA levels impairs consolidation of fear memory in mice^14, 15^. In these studies the elevation in miRNA levels and the subsequent decrease in the levels of targeted mRNA and proteins occurs immediately (< 2 h) after conditioning. In the honeybee, visual pattern learning likewise induces changes in the level of distinct miRNAs directly after conditioning^16^, suggesting that also in this organism learning triggers specific miRNA-mediated processes that contribute to memory formation. This notion is substantiated by transient blocking of miR-932 and miR-210 by miRNA inhibitors before appetitive olfactory conditioning that impairs long-term memory in honeybees^17^. Thus, studies in different species demonstrate a critical role of miRNA-dependent processes in associative learning and point to specific contributions of distinct miRNAs regarding different memory phases. However, all previous studies on the role of miRNA in associative learning focussed on long-term memory, leaving unclear whether miRNA function is also required for early memory phases.

We recently showed that learning-induced histone modifications that are epigenetic regulators of gene expression modulate early memory phases in honeybees^18, 19^. This prompted us to address the question whether miRNAs as epigenetic regulators of translation processes also contribute to early and transient memory. We explicitly analysed the function of selected miRNAs on early memory phases using the associative olfactory conditioning paradigm in honeybees^20^. This well-established paradigm takes advantage of the proboscis extension reflex (PER), elicited by a sucrose-reward, and provides the opportunity to induce a transient or a stable memory depending on the conditioning strength^21^. One-trial conditioning induces a transient memory that is insensitive to translation and transcription blockers, while 3-trial conditioning leads to a stable long-lasting memory that is impaired by translation and transcription blockers^22, 23^.

We used anti-miRNA oligonucleotides (AMO) to inhibit the function of distinct miRNAs during different phases of learning and memory. Based on sequence-specific interaction, AMOs bind to defined miRNAs and thus prevent their interaction with target mRNAs. This approach is well established and has been successfully used in cell culture studies and in *in vivo* studies in various invertebrate species, mammals, even in primates^8, 17, 24–27^. For our analysis we selected three different miRNAs miR-12, miR-124 and miR-125 that are all engaged in learning and memory and play roles in synapse-specific plasticity^3, 8, 16, 28^. Their presence in the honeybee has been proven^29^.

## Results

### Inhibitors of the miRNAs neither affect gustatory sensitivity nor non-associative learning

**Figure 1:**
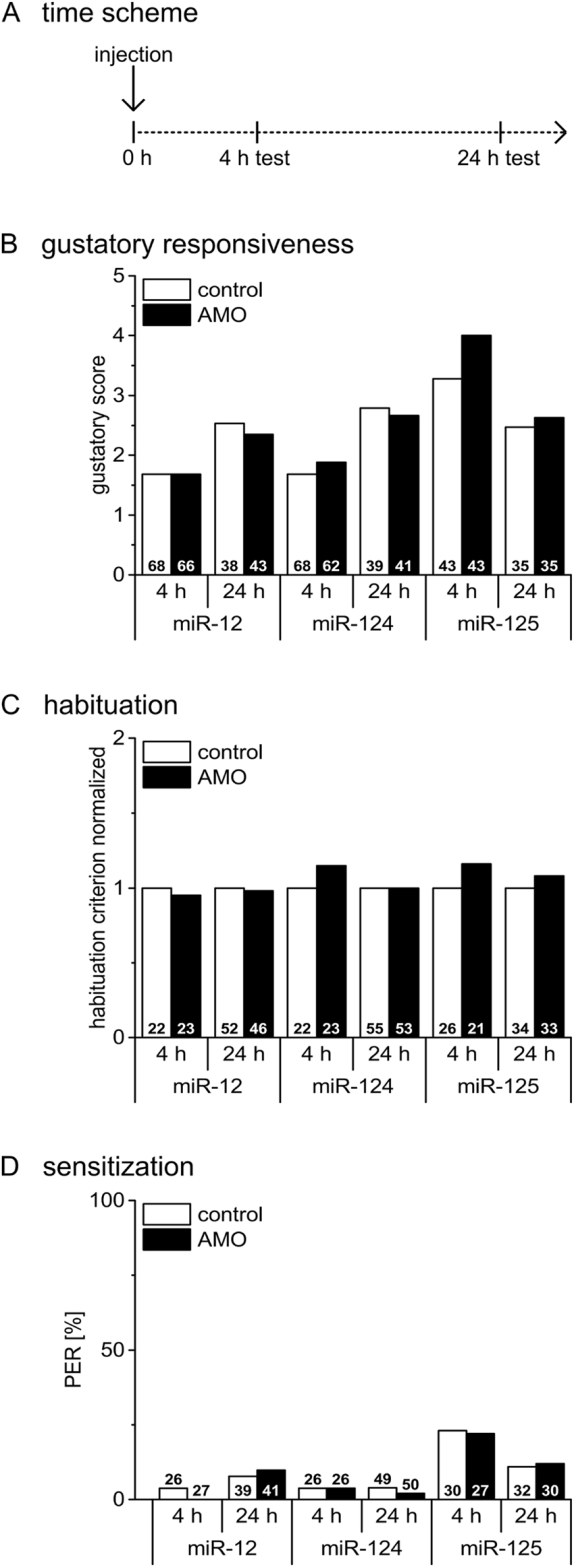
Inhihition of miRNAs neither affect gustatory sensitivity nor non-associative learning. (**A**) Four hours or 24 h after injection (miRNA inhibitors (AMO) for miR-12, miR-124 and miR-125 or the corresponding control) gustatory responsiveness, habituation and sensitization were tested. (**B**) The gustatory response scores do not differ between AMO- injected groups and their corresponding control (Mann-Whitney test; all p values > 0.3). (**C**) The habituation criterion of AMO injected groups do not differ from their corresponding control groups (Student’s t-test, all p values > 0.4). Habituation criteria were normalized to their corresponding control, each. (**D**) Sensitization as presented by the percentage of animals that show a PER after the sensitizing stimulus do not differ between the AMO-injected group and their corresponding control groups (Chi-square/Fisher exact test, all p values > 0.4). The numbers of bees tested in the different groups are indicated in each column.

To specify the contribution of the three selected miRNAs in learning processes we applied specific miRNA inhibitors (AMO) to interfere with miRNA function *in vivo*. Due to their nuclease resistance and high binding affinity, chemically modified miRNA inhibitors have been successfully fed or administered systemically into blood or tissues both in mammals and insects^17, 24, 25, 27^. Since miRNAs can potentially target different processes during the time course of learning and memory formation we injected the miRNA inhibitors either 4 h before or 1 h after conditioning. With this strategy we aim to identify the contribution of different miRNAs to early memory phases, a topic that has not been addressed so far. To exclude potential non-associative effects, we first tested whether the miRNA inhibitors affect gustatory sensitivity or non-associative learning as habituation and sensitization 4 h or 24 h after injection (Fig. 1A). The gustatory sensitivity of groups injected with miRNA inhibitors miR-12, miR-124 or miR-125 do not differ from their corresponding control groups (Fig. 1B). The same was observed for habituation (Fig. 1C) and sensitization (Fig. 1D). Since the behavioural tests are done with parallel handled groups (AMO and control) on different dates the performance can vary between the tested time points. Taken together, we found no evidence that miRNA inhibitors affect sensory processing of gustatory stimuli used as reward stimulus in associative conditioning. Moreover, miRNA inhibitors have no effect on habituation or sensitization 4 h or 24 h after injection.

### Inhibition of miR-12 or miR-124 but not of miR-125 affects early memory phases

After excluding effects of the different miRNA inhibitors on sensory processing of gustatory stimuli and non-associative learning in a time window up to 24 h after injection we addressed whether inhibition of the distinct miRNAs has an impact on early memory phases. While 1-trial conditioning induces a transient memory with a noticeable decay within 24 h, 3-trial conditioning induces a stable memory with similar PER as tested at 2 h and 24 h after conditioning. Previous studies demonstrate that the underlying molecular processes differ depending on the number of conditioning trials, even in early phases^21, 22^. Of the tested memories, only memory 24 h after 3-trial conditioning requires translation-dependent processes^22^ and is therefore considered as potential target of miRNA action. There is presently no information regarding parameters like delay or duration of miRNA inhibitor action after systemic injection. By injecting the miRNA inhibitors 4 h prior to conditioning we aim to interfere with miRNA-regulated molecular processes triggered by conditioning^21, 22^. Latter processes occur in a short time window after conditioning and comprise the presently known targets of miRNA action in synaptic plasticity^6^. Injection of miRNA inhibitors 1 h after conditioning is selected to interfere with miRNA-mediated molecular processes that occur later than 1 h after associative conditioning.

**Figure 2:**
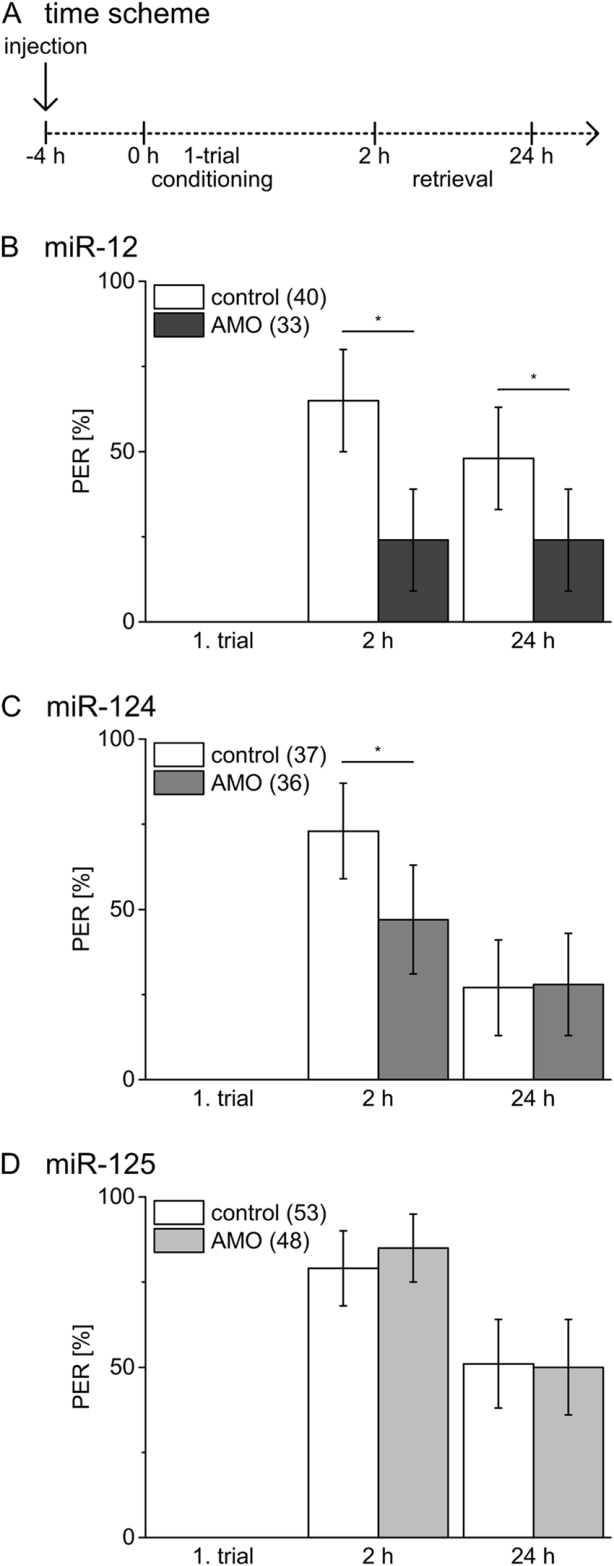
Inhibition of miRNAs before 1-trial conditioning has distinct effects on early memory phases. (**A**) Four hours before 1-trial conditioning bees were injected with inhibitors (AMO) for miR-12 (**B**), miR-124 (**C**), miR-125 (**D**) or PBS (control). Columns show the percentage of bees reacting with PER to odour presentation during conditioning and retrieval tests 2 h and 24 h after conditioning. The error bars indicate 95% binominal confidence interval and the numbers of tested animals per group are indicated in brackets. Asterisks show significant differences between groups (Chi-square/Fisher exact test (two-tailed) p < 0.05 details in Results).

**Figure 3:**
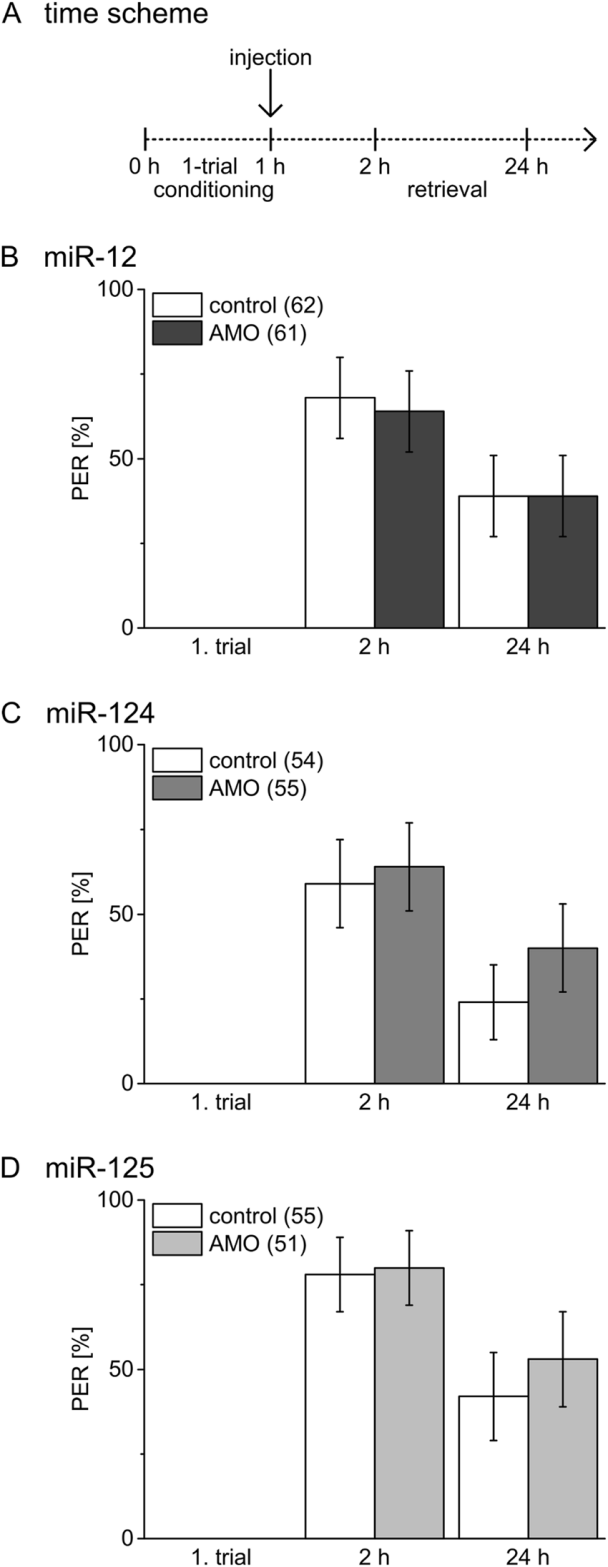
Inhibition of miRNAs after 1-trial conditioning does not affect early memory. (**A**) Bees were injected 1 h after 1-trial conditioning with inhibitors (AMO) for miR-12 (**B**), miR-124 (**C**), miR-125 (**D**) or PBS (control). Columns show the percentage of bees reacting with PER to odour presentation during conditioning and retrieval tests 2 h and 24 h after conditioning. The error bars indicate 95% binominal confidence interval and the numbers of tested animals per group are indicated in brackets. There were no significant differences (Chi-square/Fisher exact test (two-tailed) p > 0.05 details in Results).

Injection of miR-12 inhibitor 4 h before 1-trial conditioning (Fig. 2A) results in a reduction in early memory tested 2 h and 24 h as compared to control bees (Fig. 2B, X² test, 2 h: *P* = 0.008, 24 h: *P* = 0.05). Inhibition of miR-124 in the same time window results in a decrease of memory performance at 2 h, while memory tested at 24 h is indistinguishable from the control group (Fig. 2C, X² test, 2 h: *P* = 0.03, 24 h: *P* = 1). In contrast, inhibition of miR-125 has no effect on memory induced by 1-trial conditioning (Fig. 2D, X² test, 2 h: *P* = 0.44, 24 h: *P* = 1). Injection of the miR-12, miR-124 and miR-125 inhibitors 1 h after 1-trial conditioning has no effect on early memory phases (Fig. 3B, X² test, 2 h: *P* = 0.71, 24 h: *P* = 1; Fig. 3C, X² test, 2 h: *P* = 0.69, 24 h: *P* = 0.10; Fig. 3D, X² test, 2 h: *P* = 0.81, 24 h: *P* = 0.33). This strongly indicates that miR-12 and miR-124 act positively on molecular targets during and shortly after 1-trial conditioning. The different effects on memory tested 24 h after 1-trial conditioning indicates that miR-12 and miR-124 act on different molecular targets.

**Figure 4:**
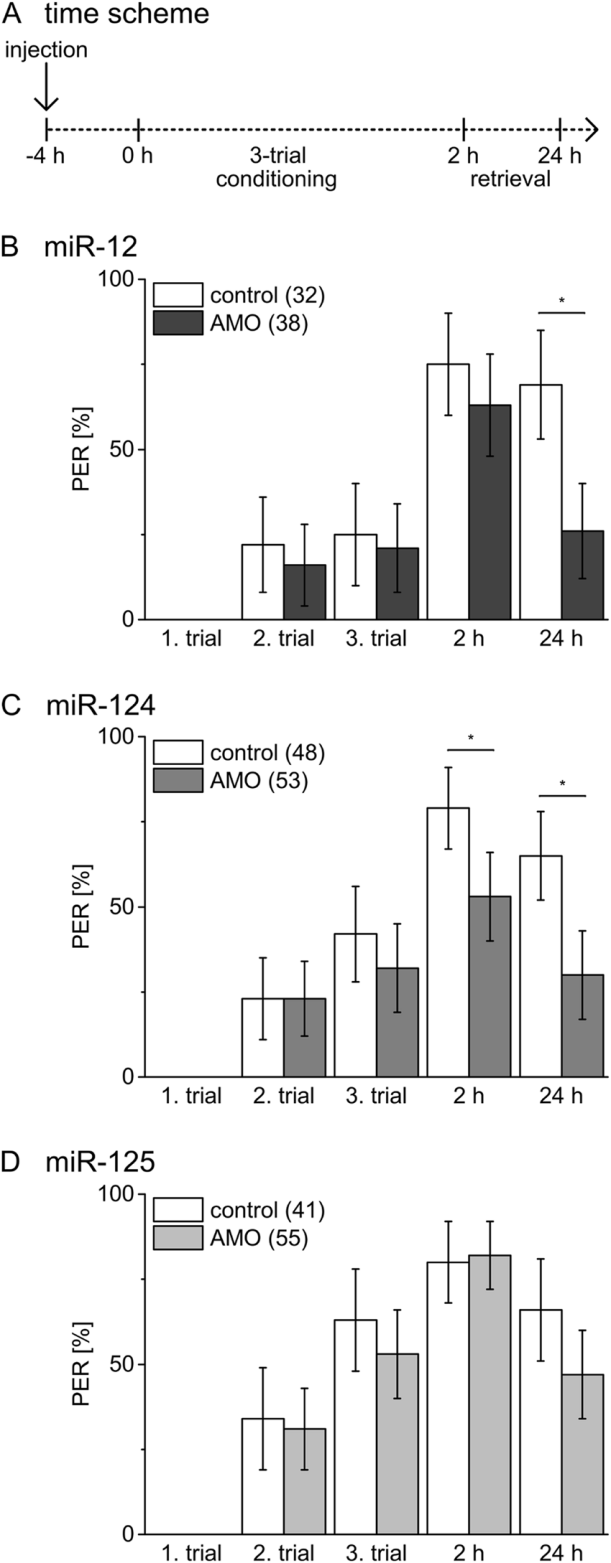
Inhibition of miRNAs before 3-trial conditioning affects early memory phases. (**A**) Four hours before 3-trial conditioning bees were injected with inhibitors (AMO) for miR-12 (**B**), miR-124 (**C**), miR-125 (**D**) or PBS (control). Columns show the percentage of bees reacting with PER to odour presentation during conditioning and retrieval tests 2 h and 24 h after conditioning. The error bars indicate 95% binominal confidence interval and the numbers of tested animals per group are indicated in brackets. Asterisks show significant differences between groups (Chi-square/Fisher exact test (two-tailed) p < 0.05 details in Results).

**Figure 5:**
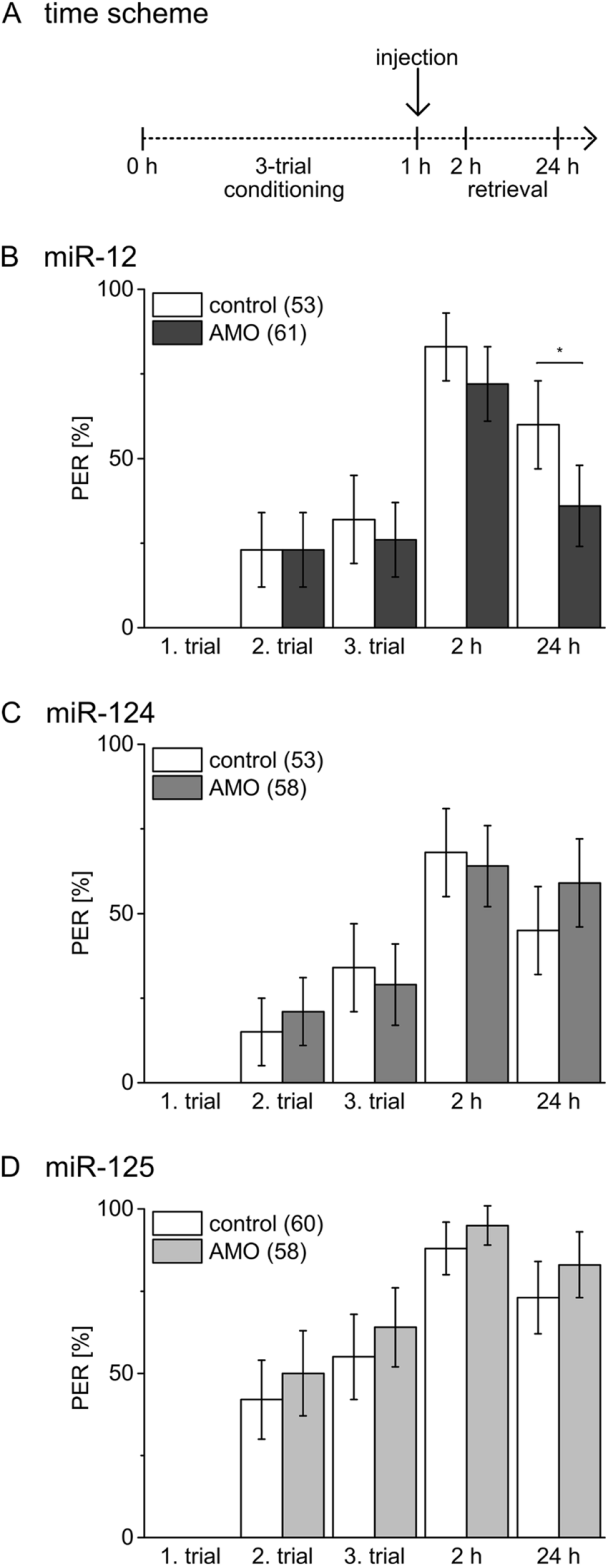
Inhibition of miR-12 after 3-trial conditioning affects memory at 24 h. (**A**) Bees were injected 1 h after 3-trial conditioning with inhibitors (AMO) for miR-12 (**B**), miR-124 (**C**), miR-125 (**D**) or PBS (control). Columns show the percentage of bees reacting with PER to odour presentation during learning and retrieval tests 2 h and 24 h after conditioning. The error bars indicate 95% binominal confidence interval and the numbers of tested animals per group are indicated in brackets. Asterisks show significant differences between groups (Chi-square/Fisher exact test (two-tailed) p < 0.05 details in Results).

To address the question whether the function of miRNAs in early memory phases depend on conditioning strength we used 3-trial conditioning that induce stable LTM in honeybees^21^. If injected 4 h before 3-trial conditioning, the miR-12 inhibitor causes a reduction of memory tested at 24 h while memory after 2 h is unaffected (Fig. 4B, X² test, 2 h: *P* = 0.31, 24 h: *P* = 0.0006). Injecting miR-124 inhibitor 4 h before 3-trial conditioning affects memory at both time points tested (Fig. 4C, X² test, 2 h: *P* = 0.007, 24 h: *P* = 0.0006). As in case of 1-trial conditioning, inhibition of miR-125 does not affect early memory as compared to the control (Fig. 4D, X² test, 2 h: *P* = 1, 24 h: *P* = 0.0969). Only bees injected with miR-12 inhibitor 1 h after 3-trial conditioning showed an impaired memory after 24 h (Fig. 5B, X² test, 2 h: *P* = 0.18, 24 h: *P* = 0.007). We found no indication that functional blocking of miR-124 (Fig. 5C, X² test, 2 h: *P* = 0.69, 24 h: *P* = 0.19) or miR-125 (Fig. 5D, X² test, 2 h: *P* = 0.32, 24 h: *P* = 0.27) after conditioning affects memory induced by 3-trial conditioning.

The findings above demonstrate that the chosen time points of injection enables a separation of miR action and support the notion of temporally restricted but different functions of miRNAs during and shortly after conditioning. For this reason we propose that the injection of the miR-12 inhibitor 1 h after 3-trial conditioning (Fig. 5B) interferes with molecular processes required for the formation of the 24 h memory > 1 h after conditioning. Consequently, the latter targets of miR-12 most likely differ from the targets during or shortly after conditioning (Fig. 4B).

## Discussion

**Figure 6:**
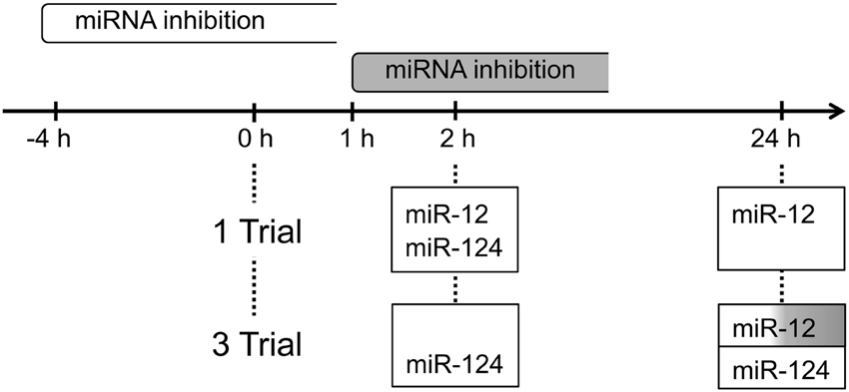
Scheme illustrating the contribution of different miRNAs to early associative memory phases in honeybees. The scheme visualizes the different time windows of miRNA inhibition. The inhibitors were either injected 4 h before (white) or 1 h after (grey) conditioning. The miRNAs that contribute to distinct early memory phases induced by either 1-trial or 3-trial conditioning are indicated in the boxes. White boxes indicate the requirement of miRNA action in the time window 4 h before to at least 1 h after conditioning. Grey shading indicates the requirement of miRNA action in a time window starting from 1 h after conditioning.

Our comparative approach using olfactory conditioning in the honeybee reveals first evidence that miRNAs are also involved in the formation of early memory phases that have been considered as insensitive to translation blockers. Using miRNAs inhibitors we show that miR-12 and miR-124 differ regarding their contribution to early memory phases, while miR-125 seems to be not involved in early memory formation at all. We conclude that miR-12 and miR-124 are both positive regulators required during and/or shortly after conditioning, while function of miR-12 is also required > 1 h after 3-trial conditioning (Fig. 6).

Until now, studies on the role of miRNA as regulators of translation focussed on long-lasting neuronal plasticity and formation of LTM. Regulation of miRNA levels by learning and neuronal plasticity has been reported in invertebrate and mammalian nervous systems. Induction of fear memory in mice^13–15, 30^, learning in honeybees^16, 17, 31^, or induction of long-term potentiation (LTP) in mice^32, 33^ lead to immediate changes in miRNA levels. Although the dynamics of the changes in miRNA levels differ between the experimental setups the interaction of the miRNAs with their target mRNAs always regulate translation processes and thus modulate synaptic plasticity or formation of memories. The different *in vitro* and *in vivo* studies demonstrate that miRNAs target mRNAs that are well known key components of synaptic release, receptor composition, transcription factors, and spine growth^2, 33, 34^.

While the signalling processes that regulate the levels of distinct miRNAs in the different neuronal compartments have not been characterized in detail, the molecular processes that mediate biosynthesis and degradation of miRNAs are well described and provide targets for learning-induced regulation of miRNA levels^35–37^. The enzymes that are responsible for synthesis and degradation of miRNAs are located in the soma but part of this machinery is placed in dendrites where it contributes to synapse specific plasticity^34, 36^. Very recent observations added an additional level of complexity by demonstrating that distinct precursor miRNAs (pre-miRNAs) and mature miRNAs are specifically localized in defined compartments within neurons^38–40^. This raises the question whether the translation blockers used to characterize translation-dependent memory phases in honeybees^22, 23^ block translation processes in all cellular compartments. It is feasible that translation blockers do not efficiently inhibit miRNA-regulated translation in all of the cellular compartments mentioned above.

Although the targets of the miRNAs contributing to early memory formation are unknown, several studies show that miRNA-mediated processes lead to changes at the protein level that can be observed within 1-2 h. As an example of a local mRNA regulation it has been shown that activation of hippocampal neurons can trigger degradation of the MOV10 protein that is a component of the RISC complex in synapses^41^. The rapid decay of MOV10 (half-time ≈8 min) releases translation suppression by miRNAs and thus elevates protein levels in synapses within 1 h. In case of fear memory, conditioning induces a fast elevation of miRNAs followed by down-regulation of the target proteins within 2 h^14, 15^. Thus, the observed effects of miRNA inhibitors on early memory tested 2 h after conditioning in honeybees are realistic at the molecular level. However, the identification of the mRNA targets that contribute to distinct memory phases is challenging since each single miRNA can target many different mRNAs^2, 33, 34^.

The molecular processes underlying memory formation by 3-trail conditioning have been well described and clearly differ from memory induced by 1-trial conditioning^18, 19, 21, 22, 42^. However, the knowledge is biased towards the stable long-lasting memory induced by 3-trial conditioning. In honeybees an early memory phase (2-24 h) induced by 3-trial conditioning requires Ca^2+^-dependent activation of calpain that cleaves protein kinase C into the constitutively active protein kinase M^22^. Thus it is feasible that the calpain-mediated activation of Dicer and thus the up-regulation of miRNA synthesis^43^ is specific to 3-trial conditioning. Future investigations will have to verify whether 3-trial conditioning activates Dicer and thus affects the level of miRNAs in honeybees.

The finding that miRNAs also regulate immediate and transient memories demands a more detailed characterization of miRNA requirement in memory formation with respect to time windows. Moreover techniques that allow local and transient inhibition of miRNA function in distinct brain areas will be helpful to elucidate the dynamic aspects of miRNA function in formation of both, stable LTM and transient memories.

## Materials and Methods

### Animals

Honeybees (*Apis mellifera*) were from the apiary of the Saarland University (Campus Saarbrücken, Germany). In summertime the honeybees were collected from hives located in the botanical garden. Foragers were caught in front of the hive with a UV light-permeable Plexiglas pyramid and transferred into plastic vials. In wintertime the honeybees were kept indoors and caught directly into plastic vials. Bees were cold-immobilized and mounted in plastic tubes wherein they were able to move antennae and mouthparts. In the evening, the bees were fed with 1 M sucrose solution until satiation. Throughout the experiment, bees were kept in a dark chamber at 20-25 °C and 70% relative humidity. Repetitions of experiments were carried out with bees from different hives to avoid hive-specific effects.

### Drug application

The thoracal tergum of the honeybee was pricked with a fine cannula and 1 µl of the substance was injected with a calibrated glass capillary into the haemolymph. Animals were injected with 0.5 µM miRNA inhibitor designed after Lennox *et al*.^44^ and purchased from Integrated DNA Technologies (Coralville, Iowa, USA). The applied inhibitors, anti-ame-miR-124 (#66477088), anti-ame-miR-12 (#66388547) and anti-ame-miR-125 (#68529874) were diluted in PBS (137 mM NaCl; 2.7 mM KCl; 10.1 mM Na_2_HPO_4_; 1.8 mM KH_2_PO_4_).

### Behavioural analysis

For behavioural tests, bees were caught the day before the experiments and mounted as described above. Behavioural experiments were performed as described by Merschbaecher *et al*.^18, 19^. Bees were injected at the time-points indicated in the results section.

The responsiveness to gustatory stimuli was tested by monitoring the PER after touching the antennae with toothpicks, moistened with sucrose solutions. Stimuli were applied with 2 min inter-stimulus interval, starting with water (0 M sucrose) continuing with increasing sucrose concentrations (0 M, 0.03 M, 0.1 M, 0.3 M and 1 M). The gustatory response score (0–5) of individual animals represents the sum of PER elicited by the five gustatory stimuli.

Habituation to sucrose stimuli was tested by repeated stimulation of one antenna with 1 M sucrose solution with a 1 s inter-stimulus interval. Animals were considered habituated, when the PER did not occur anymore for five times in a row and animals elicited a PER to a consecutive stimulus to the contralateral antenna (dishabituation). The number of stimuli applied until habituation was noted for evaluation. Bees that showed more than 50 PERs (< 7%) and bees that did not show a PER to the dishabituating stimulus were excluded from the evaluation of data (< 8%).

Before sensitization, hungry bees were stimulated with an odour stimulus (clove oil) to test for spontaneous response to odour. After 2 min an appetitive stimulus (1 M sucrose) was presented to the antennae of the bees, followed 20 s later by presentation of the odour stimulus. Bees are considered as sensitised when they respond to the odour presented after the strong sucrose stimulus. Animals that did not react to the sucrose stimulus were excluded from evaluation (< 4%).

For associative olfactory conditioning, honeybees were starved over night for at least 16 h. An acquisition trial consists of an odour stimulus (CS, conditioned stimulus) followed by a sucrose reward (US, unconditioned stimulus). The CS (clove oil) was presented for 5 s and 3 s after onset of the odour the US was presented to the antennae followed by feeding the bees for 3 s with a toothpick moistened with sucrose. A single CS-US pairing represents a weak conditioning while three CS-US pairings applied with a 2 min inter-trial interval represent a strong conditioning. In the stimulus-control groups (unpaired) the honeybees received the CS and the US stimuli separated by 15 s. Memory was tested after 2 h and 24 h by presentation of the CS alone. Animals not responding to the US during the conditioning were excluded from the experiments (< 10%).

### Statistical Analysis

Statistical analysis was conducted by using http://vassarstats.net/. Comparison of behavioural data (PER) was carried out with the Chi-Square/Fisher’s exact test. The Yates value and the two-tailed Fisher’s exact probability value for each comparison are presented, whereby *P* < 0.05 was considered as significant. The gustatory response scores were tested by Mann-Whitney U-test.
